# Nostalgic conversations: The co-production of an intervention package for people living with dementia and their spouse

**DOI:** 10.1177/14713012211047350

**Published:** 2021-10-08

**Authors:** Emily Dodd, Sanda Ismail, Gary Christopher, Tim Wildschut, Constantine Sedikides, Richard Cheston

**Affiliations:** Department of Health and Social Sciences, 14262 University of the West of England, Bristol, UK; Department of Psychology, 7423 University of Southampton, Southampton, UK; Department of Health and Social Sciences, 1981 University of the West of England, Bristol, UK

**Keywords:** Alzheimer’s disease, nostalgia, self-esteem, self-growth, meaning in life, social connectedness

## Abstract

**Objectives:**

Nostalgic memories are more social than other forms of autobiographical recall, often refer to atypical events, express more positive affect and reflect life as meaningful. Recalling a nostalgic (compared to ordinary) memory increases self-esteem, self-growth, meaning in life and social connectedness for people living with dementia. We set two objectives: to work with people living with dementia to develop an intervention based on nostalgia, and to assess whether couples could engage in nostalgic conversations.

**Method:**

Our research fell into three phases. Initially, we consulted with people living with dementia and with carers to identify the parameters for a nostalgic intervention. From this, we drafted a workbook that contained triggers for nostalgic conversations, which we then took back to the public contributors for refinement. Finally, we trialled the workbook over 5 weeks with six couples, each of which included a person living with dementia. We assessed pre- and post-intervention self-esteem, self-growth, meaning in life and social connectedness for participants with dementia and social connectedness for carers. We then calculated Reliable Change Index scores and established levels of clinically significant change. We also interviewed couples at the end of the intervention to explore its implementation and acceptability.

**Results:**

All six couples could identify nostalgic memories, with five couples successfully integrating the nostalgic conversations into their day-to-day lives. A sixth couple found it difficult to engage fully with the intervention, but still considered it useful. All six couples manifested a reliable change in at least one outcome, with one couple showing reliable change across three outcomes.

**Conclusion:**

The psychological benefits of nostalgia have been robustly demonstrated in laboratory-based studies. This co-production of an intervention that sets nostalgic recall into the context of a conversation has clinical potential but requires further investigation through a larger study.

## Introduction

The New Oxford Dictionary of English (1998, p. 1266) defines nostalgia as a ‘sentimental longing or wistful affection for the past’. Nostalgia refers to the evocation of personally relevant and emotionally poignant memories in which the self plays a prominent role within a social context (Sedikides et al., 2008, 2015). The emotion occurs frequently (e.g. several times a week; Hepper et al., 2021; Wildschut et al., 2006), and across ages (Madoglou et al., 2017; Turner & Stanley, 2021) and cultures (Hepper et al., 2014; Wildschut et al., 2019). Research on adults without a cognitive impairment has shown that when a person nostalgises, they feel content, comforted and often joyful, but also experience a tinge of yearning or sadness for the irrevocability of the valued events; as such nostalgia is a bittersweet emotion, albeit more sweet than bitter (Leunissen et al., 2021; Sedikides & Wildschut, 2018).

Nostalgia has been intensely researched over the past 20 years in social psychology. In relevant experiments, participants reflect on a nostalgic event from their lives (compared to an ordinary or positive event). The literature has established that the emotion serves self-oriented, existential and social benefits (Sedikides et al., 2015; Wildschut & Sedikides, 2020). For example, in terms of self-oriented benefits, nostalgia (vs. a control group) heightens self-esteem (Wildschut et al., 2006) and augments self-growth (i.e. the subjective perception of ‘the potential to cultivate inner potentialities, seek out optimal challenges and integrate new experiences into the self-concept’; Baldwin & Landau, 2014, p. 162). In terms of existential benefits, nostalgia raises meaning in life (i.e. the perception that life is purposeful; Sedikides & Wildschut, 2018). Lastly, in terms of social benefits, nostalgia fosters social connectedness (i.e. a sense of belongingness and acceptance including being loved, protected and supported; Sedikides & Wildschut, 2019).

Nostalgia’s psychological benefits are not confined to the general population. Three experiments by Ismail et al. (2018) showed that people with mild or moderate levels of dementia reaped similar benefits. Experiment 1 tested whether recall of a nostalgic (compared to an ordinary) event enhanced psychological benefits, with participants in the nostalgia arm reporting significantly higher levels of self-esteem, meaning in life and social connectedness. To establish convergent validity, Experiment 2 used an alternative method of nostalgia induction. Participants were recruited into the experiment in pairs, with both members of a pair being asked to provide a favourite nostalgic song. One member was then randomly assigned to the nostalgia arm and the other to the control arm. The former listened to one of their favourite, nostalgic songs. The identical song was also played to the yoked participant in the control arm. Thus, whereas both participants listened to the same piece of music, it had only been identified as a nostalgic trigger for one of them. Results were similar to those of Experiment 1, with participants in the nostalgia (compared to control) arm reporting higher self-esteem, meaning in life and social connectedness.

In Experiment 3, Ismail et al. (2018) tested whether the psychological benefits of nostalgia would act to improve recall and recognition of dementia-related information. Participants were randomised either to a nostalgia or control condition using the same procedure as in Experiment 1. They were then read a list of 24 dementia-related statements, which they imagined as relevant to themselves. Twelve statements had previously been rated as negative (e.g. ‘*My illness means that I may forget the names of friends or family*’) and 12 as positive (e.g. ‘*Even with the illness I can be reassured*’). Afterwards, participants recalled as many of the 24 statements that they had just heard as they could. Compared to those in the control condition, participants in the nostalgia condition reported higher self-esteem, meaning in life and social connectedness.

Additionally, an analysis by Ismail et al. (2021) of recordings of nostalgic and ordinary memories produced by people living with dementia indicated that, as in the general population, the content of nostalgic memories among people with dementia is predominantly social (Wildschut et al., 2006, 2018). Specifically, compared to ordinary autobiographical memories (i.e. memories for specific episodes and conceptual, generic or schematic knowledge about the person’s life; Conway and Rubin, 1993), nostalgic memories featured a higher proportion of social words (especially words representing close figures from the person’s past such as ‘mother’, ‘father’ or ‘friend’) and contained higher levels of companionship, affiliation and close relationships.

## Nostalgia’s potential as a clinical intervention

To date, nostalgia has largely been experimentally induced in the general population under controlled conditions and for a relatively brief period. As of late, however, nostalgia’s potential as an intervention that contributes to psychological well-being over the longer-term is increasingly being explored. A trial of a 6-week nostalgia intervention with 176 undergraduate students randomised either to a nostalgia or a control condition demonstrated increases in well-being for nostalgic participants (Layous et al., 2021), with nostalgic reflection being beneficial to the degree that it fostered meaning in life and social connectedness. In addition, a nostalgic digital application has helped older people to learn to use new technology in a more intuitive way (Bong et al., 2020), whilst a nostalgic remembering intervention is being developed to strengthen feelings of safety and to promote adaptive physiological and psychological regulation amongst dyads receiving palliative care for heart failure (Fleury et al., 2021).

Although this recent exploratory research suggests that nostalgia has intervention potential, the neurological impact of dementia on a person’s cognitive abilities, including their ability to access autobiographical memories (Morris & Mograbi, 2013), may reduce nostalgia’s intervention efficacy. That is, the more limited access to autobiographical memories may diminish a person’s sense of self (El Haj et al., 2015a) – their ability to define themselves in relation to important others (El Haj et al., 2015a) and to maintain a sense of connection between their past and present (Ben Malek et al., 2018; El Haj et al., 2015b). Regardless, we opted to explore the promise of an intervention among persons living with dementia, given nostalgia’s capacity to boost, at least momentarily, psychological benefits in this population (Ismail et al., 2018). The current study, therefore, had two objectives: (i) to co-produce a nostalgia-based intervention with people living with dementia and their carers; and (ii) to test the acceptability and uptake of the intervention by trialling it with people living with dementia and their partner.

## Materials and method

We detail the results of a preliminary three-phase effort towards realising our objectives. In the first stage, we worked with people living with dementia and carers to co-produce a workbook of nostalgic conversations. We then refined this with the help of people with lived experience of dementia. Finally, we trialled the workbook with six couples over a 5-week period, with a nostalgia coach offering regular support to the couples.

### Co-production of the nostalgia intervention

Two researchers (ED and SI) sought out public contributors by distributing advertising materials through local dementia networks and at family health centres. In all, we arranged five face-to-face meetings (one group session, three individual meetings and one meeting with a married couple) with eight public contributors. Two public contributors were living with dementia, while the other six had all provided care to relatives. One carer was from the Chinese community with all other participants being white-British. We offered public contributors a shopping voucher in recognition of their time to the project.

The public consultation focused on three areas: participants’ feelings about nostalgia in general; circumstances in which nostalgia might be useful; and possible core features of an intervention. The consultation process confirmed that being nostalgic is regarded as a valuable emotion, identified ways in which nostalgia could be triggered and developed the principles to be used at an intervention. Four principles emerged: (i) the intervention ought to consist of individualised, home-based sessions; (ii) the intervention should be straightforward, so that carers could implement it with a minimum of training and support; (iii) the intervention must be incorporated into the day-to-day lives of persons living with dementia rather than being an additional task that required extra time; and (iv) the assessment ought to be attentive to the possibility of discomfort among persons who have had traumatic memories. In consultation with TW and CS, ED and SI then drafted a workbook setting out how couples could integrate nostalgia into their conversations. The workbook was designed to be completed by a couple over a 5-week period, with weekly support being provided either through face-to-face visits or over the telephone. The workbook therefore acted as a record of the couples’ own memories and triggers to which they could refer during the week and as a guide for resolving barriers to engagement. Subsequently, public contributors commented on the workbook prior to it being trialled.

### Nostalgic conversations

The intervention that emerged from this consultation process lasts for five weeks, with participating couples being supported by nostalgic coaches (ED and SI) through alternating weekly home visits and phone calls. During the first appointment, coaches introduce the workbook and encourage couples to (i) identify their nostalgic memories as well as the potential triggers of those memories (e.g. photos, places and music), and (ii) describe these memories in writing in the workbook. The workbook serves as a guide for both the person with dementia and their partner and includes instructions aimed to facilitate the elicitation of nostalgic memories (i.e. immersion into the pertinent nostalgic event). The coaches then plan with the couple how to incorporate nostalgic conversations into the couple’s daily routine over the following week. Couples were encouraged to identify any potential barriers they might encounter during the week that could prevent them from using the intervention. Couples were instructed to record in their workbook any implementation of the intervention during the five weeks. At each follow-up session, coaches used the information written in the workbook as the basis for discussion.

### Participants and design

The study received ethical approval^1^. Couples were eligible for inclusion, if one member: (i) had a diagnosis made within the previous 24 months of probable Alzheimer’s disease according to the NINCDS ADRDA criteria (McKhann et al., 2011), probable Vascular Dementia according to the NINDS AIREN criteria (Román et al., 1993) or a mixed form of these; (ii) had a mild to moderate level of cognitive impairment (e.g. an MMSE score of 15 or over; Folstein et al., 1975); (iii) had the capacity to consent; and (iv) had sufficient communication skills to take part in the research. Carers were eligible, if they had capacity to consent and sufficient communication skills to take part in the research. We excluded couples if either partner disclosed a history of past trauma. Between October 2018 and January 2019, we recruited six husband-and-wife couples living together (12 participants, all white-British) through an independent memory clinic for NHS patients. We provide details about participants in Table 1, and information about the intervention in Table 2.

**Table 1.** Demographic information about the participants.

Couple	Person living with dementia (age)	Diagnosis	Time since diagnosis (months)	MMSE^a^	Carer – relationship (age)
1	Peggy (74)	Mixed dementia	16	18	Keith – husband (74)
2	Linda (80)	Alzheimer’s disease	19	25	Bob – husband (84)
3	Mary (72)	Alzheimer’s disease	18	15	George – husband (75)
4	Tom (84)	Vascular dementia	2	23	Gwen – wife (84)
5	Harry (84)	Alzheimer’s disease	16	24	Joan – wife (85)
6	Audrey (83)	Vascular dementia	1	23	Jack – husband (86)

^a^MMSE scores were assessed using ‘WORLD Backwards’ option. All names are pseudonyms.

**Table 2.** Information about the type and length of each session across the six couples.

	Average length of each session in minutes (range)	Number of sessions delivered	
		Face to face	Phone
Session 1 (includes collecting consent and preliminary outcome measure data)	114 (75–240)	6	0
Session 2	14 (2–50)	1	5
Session 3	30 (15–60)	2	4
Session 4	25 (15–60)	1	5
Session 5 (includes collecting follow-up outcome measure data and conducting acceptability interview	74 (60–105)	6	0

### Outcomes measures

Previous research has established that nostalgia serves self-oriented, existential and social functions (Sedikides et al., 2015; Wildschut & Sedikides, 2020). We assessed each of these three domains at baseline and again at a five-week follow-up appointment. To assess the self-oriented domain, we measured self-esteem (Wildschut et al., 2006) and self-growth (Baldwin & Landau, 2014). We assessed the existential domain by measuring meaning in life (Sedikides & Wildschut, 2018). Lastly, we assessed the social domain by measuring social connectedness (Sedikides & Wildschut, 2019). Participants who were living with dementia completed the following instruments:

1. Self-esteem. The Rosenberg Self-Esteem scale (RSES; Rosenberg, 1989) asks participants to rate 10 statements on a 4-point scale (0 = strongly disagree, 3 = strongly agree). A sample item is: ‘I feel that I’m a person of worth, at least on an equal basis with others’. The RSES has been used in research with people living with dementia (Lamont et al., 2020; Marshall et al., 2015).
2. Self-growth. The 7-item Personal Growth subscale of the Psychological Well-being scale (PWB scale; Ryff, 1989) includes items such as: ‘For me, life has been a continuous process of learning, changing and growth’ (1 = strongly disagree, 6 = strongly agree). The PWB scale has been used in dementia research (Gonzalez et al., 2015).
3. Meaning in life. The 7-item Purpose in Life subscale of the PWB scale includes item such as: ‘I have a sense of direction and purpose in life’ (1 = strongly disagree, 6 = strongly agree).
4. Social connectedness. The 7-item Positive Relations with Others subscale of the PWB scale includes items such as: ‘I enjoy personal and mutual conversations with family members and friends’ (1 = strongly disagree, 6 = strongly agree).

Carers rated their level of social connectedness with the care recipient (i.e. the person living with dementia) using the 7-item Satisfaction with the Care Recipient (SCR) subscale of the Sense of Competence Questionnaire (Jansen et al., 2007). A sample item is: ‘I feel that my …. behaves the way s/he does to have her/his own way’ (1 = *agree, very strongly*, 5 = *disagree, very strongly*). The SCR scale is specifically designed for carers of people living with dementia and has been widely used.

### Acceptability interviews

After the final session, we carried out face-to-face, semi-structured interviews with all six couples to understand how participants had experienced the research process and intervention including completing the workbook and data collection process. These interviews also probed for potential improvements to the intervention (e.g. *How easy/difficulty was it for you to identify the triggers of your nostalgic memories? How did you find the process of trying to re-live those memories using the workbook? Do you feel our follow-up process was helpful? Was it sufficient? Would you like more of that? Was the workbook format easy to use? Do you think there is a much better format or approach?*)*.* All interviews were recorded.

### Data analysis: Reliable change index/clinically significant change

We analysed change on a case-by-case basis in terms of the Reliable Change Index (RCI) and Clinically Significant Change (CSC), a technique used elsewhere to evaluate the impact of novel psychological techniques in dementia research (Craig et al., 2018). The RCI provides a standardised score representing the change in pre- and post-intervention scores divided by the standard error of the tests being used. We calculated RCI scores through an online spreadsheet^2^ using comparison data, including reliability coefficients, taken from studies with similar populations: (i) for the RSES from Marshall et al. (2015); (ii) for the SCR from Graff et al. (2006) and Jansen et al. (2007); and (iii) for the three PWB subscales from Fawn (2018). The CSC analysis defines clinical significance in terms of participants moving across a clinically significant boundary. As the measures we used do not have clinical thresholds, we followed the procedure set out by Jacobson and Truax (1991) and Jacobson et al. (1999), and established a cut-off point that was greater than or equal to 1.96 standard deviations from the mean for each variable.

## Results

### Implementation and feasibility of the intervention

The six dyads showed good engagement with the study by attending all of the sessions and managing to identify nostalgic memories and triggers for those memories. The most common method of inducing nostalgia reported by couples was looking at old photographs. Other triggers included listening to pieces of favourite music, souvenirs from travels and visiting places that were nostalgic. Typically, nostalgic memories concerned travel and holidays, work life, family life and hobbies such as playing a musical instrument or sports. Three couples (Linda/Bob, Mary/George and Harry/Joan^3^) had nostalgic conversations at least once a week and reported that they were able to integrate them into familiar conversational patterns. Another two couples (Tom/Gwen and Audrey/Jack) required assistance from the coaches, but nevertheless found the intervention helpful.

One couple (Peggy and Keith) found recalling a nostalgic past challenging. First, compared to the other couples in the study who had all been together for over 50 years, this was Peggy and Keith’s second marriage and they had been together 23 years. Consequently, when compared to other couples, they did not have a reservoir of shared memories established during their earlier adult life to draw upon. Secondly, Keith’s busy schedule and Peggy’s poor physical health meant that they had limited opportunities for talking about the past. Finally, although we included a question in our screening of potential participants asking whether they had previously experienced trauma, neither Peggy nor Keith disclosed at that point that her previous marital relationships had been abusive. It was only when they began to talk about her nostalgic memories that the distressing nature of some of these events resurfaced. The researcher offered them the option to withdraw, but they decided to continue. However, Keith was cautious about asking Peggy to think back to her past, and instead the couple visited places with nostalgic connotations in order to trigger memories and feelings of well-being. Perhaps because of these difficulties in drawing on nostalgic memories, while Peggy’s meaning in life scores improved after the intervention, her personal growth score showed deterioration. Peggy did not complete the SES and Keith did not complete the SCR after the intervention, as both stated that they found completing the measures difficult.

### Acceptability of the intervention

All couples agreed that the length and number of sessions with the researcher were appropriate. Participants also made suggestions as to how the intervention could be improved, including changes to the wording and structure of the workbook. The suggestions included structuring the workbook as a diary, making the page headings and sections clearer, moving the problem-solving section to the end of the booklet and adding sessional checks to identify how nostalgic the couple is after they have used the intervention. George (a carer) reflected that both he and Mary found that the affective boost they had gained from the nostalgic conversation lasted throughout the day. George also reported that, even though they were prone to talking about the past, the focus on regularly engaging with specific nostalgic memories which they then recorded in the workbook made him see the value in these conversations. As an illustration of this process, when he and Mary recalled dancing during their courtship, George described how this nostalgic conversation triggered them to start dancing around the living room together again.

### Psychological benefits

Table 3 shows changes across the intervention for each couple. We identified reliable change as occurring when the change between pre- and post-scores was greater than the RCI. For the RSES, this was a change of 5 points or more; for the remaining scales, this equated to pre-and post-intervention changes of 4 points or more. All six couples manifested a reliable change in at least one outcome, with one couple (Linda and Bob) showing reliable change across three outcomes (self-esteem, self-growth and carer social connectedness). One person with dementia reported a reliable improvement in self-esteem, four persons in self-growth (although a fifth, Peggy, showed a decrease) and one person in meaning in life.

**Table 3.** Pre- and post-intervention scores, reliable change and significant change.

	Peggy and Keith	Linda and Bob	Mary and George	Tom and Gwen	Harry and Joan	Audrey and Jack
Self-esteem (SES)						
Pre	22	1	27	20	30	22
Post	—	28	30	17	29	19
RCI (4.96)	—	Improve	No change	No change	No change	No change
CSC ( ≥24 )	—	YES	(yes)	NO	(no)	NO
Self-growth (PWB: Personal growth)						
Pre	42	31	36	25	29	29
Post	27	35	41	26	35	34
RCI (3.22)	Deteriorate	Improve	Improve	No change	Improve	Improve
CSC ( ≥41 )	NO	NO	YES	NO	NO	NO
Meaning in life (PWB: Purpose in life)						
Pre	28	34	39	26	36	32
Post	33	35	41	28	34	33
RCI (3.85)	Improve	No change	No change	No change	No change	No change
CSC ( ≥41 )	NO	NO	YES	NO	NO	NO
Person with dementia social connectedness (PWB: Positive relations with others)						
Pre	39	40	42	31	39	40
Post	40	38	41	35	41	40
RCI (3.15)	No change	No change	No change	Improve	No change	No change
CSC ( ≥41 )	NO	NO	(no)	NO	YES	NO
Carer social connectedness (SCR: Satisfaction with the care recipient)						
Pre	33	28	28	7	36	35
Post	—	33	26	33	35	34
RCI (3.53)	—	Improve	No change	Improve	No change	No change
CSC ( ≥31 )	—	YES	NO	YES	(no)	(no)

Note: Pre = baseline assessment; Post = 5-week follow-up; CSC: Clinically Significant Change; PWB: Psychological Well-Being scale; SES:= Rosenberg Self-Esteem scale; SCR: Satisfaction with the Care Recipient scale; RCI: reliable change index; ‘—’ denotes missing data.

YES/NO indicates that baseline scores were below the Clinically Significant Improvement threshold and whether pre-to post-intervention change did/did not cross this threshold; (yes) indicates that while there was a pre-to post-intervention improvement, the participant scored beyond the clinical threshold at baseline, so was not able to improve further; (no) indicates both that there was a pre-to post-intervention fall in scores and that the participant scored beyond the clinical threshold at baseline and follow-up.

Clinically significant improvement was taken as occurring when there was a pre-to post-intervention improvement in participants’ score on a measure that passed a threshold equivalent to or greater than 1.96 standard deviations from the mean scores provided by the comparison data (Jacobson et al., 1999; Jacobson and Truax, 1991). For participants with dementia, this was a score on the RSES of 24 (out of a maximum of 40); for self-growth, meaning in life and social connectedness, this was a score of 41 (out of 42). For carers, this was a social connectedness score of 31 (out of 35). Both partners in one couple (Tom and Gwen) evinced clinical improvement in social connectedness, with another carer (Bob) also showing evidence of clinical improvement in social connectedness.

## Discussion

### Summary of findings

This study has demonstrated that people living with mild or moderate levels of dementia and their partners can use nostalgia as part of their everyday conversations. All six couples completed the five-week intervention and showed reliable change in at least one outcome, although one participant with dementia (Peggy) reported deterioration in personal growth. Couples used an array of triggers to generate nostalgia and structured their conversations by drawing on a combination of the workbook, face-to-face and telephone support. The intervention may improve self-esteem, self-growth, meaning in life and social connectedness, with the strongest evidence for improvement being shown in enhanced levels of personal growth for the person with dementia.

As we mentioned, Peggy’s earlier life included a history of abuse – something that was not disclosed during the assessment process. In the future, we will adjust the assessment process so that we ensure that different forms of trauma, including abusive relationships, can be identified more reliably. Following feedback from participants, we will also adapt the workbook to incorporate more precise instructions to help participants distinguish nostalgia from simply remembering past events.

### The co-production process

We report the development of the nostalgic conversations’ intervention. Central to this has been the two-phase methodology in which we first drew on the expertise of people living with dementia and their carers to form a novel intervention before using field testing to refine it. Indeed, the importance of co-production has been increasingly recognised in dementia research (Hales & Fossey, 2018) as a way to combine the strengths of academic and professional expertise with the engagement of people having deep and personal knowledge of the context in which the intervention is to be implemented. This collaboration enables health care interventions to be tailored to the needs, expectations and skills of the people who will be using them (Tummers et al., 2016). The co-production process guided us to involve nostalgia in a way that was personalised and participant led. Our initial consultation showed that there was little enthusiasm for what might be thought of as a traditional model of psychological interventions – namely, structured, weekly group sessions delivered by a health care worker. Instead, the intervention that emerged from these discussions, and which was then refined through our collaborative work with six couples, was a looser process with couples jointly identifying triggers for conversations about nostalgic memories that were then incorporated into their daily routines.

### Study strengths and limitations

This study has several methodological strengths, most notably the use of a co-production approach to bring an effect established in the psychological laboratory into potential clinical practice. Nevertheless, our findings are limited. First, we exclusively recruited husband/wife couples, and so are unable to comment on whether other couple types (e.g. parent and adult children) could profit from nostalgic conversations. This is a promising direction for future research, as adult children often assume caring responsibilities for parents living with dementia. Second, when talking about the past, carers can make conversational errors that may leave the person with dementia feeling as if their memory is being tested or that they are being put on the spot, and thus inhibit discussion (Williams et al., 2019). Consequently, while further testing (e.g. of how coaches can be trained), as well as trialling of the nostalgic conversations’ intervention are called for, it is important to attend both to what is discussed when people talk about the past and how it is being discussed. Finally, the findings can be enriched by a more detailed, qualitative analysis of the way in which couples engaged with the intervention.

## Conclusion

The nostalgic memories of people living with dementia relate to momentous life events that reflect central aspects of the person’s self and their relationships with others (Ismail et al., 2021). Nostalgic (vs. control) recollections heighten self-esteem (Wildschut et al., 2006), nurture self-growth (Baldwin & Landau, 2014), increase meaning in life (Sedikides & Wildschut, 2019) and foster social connectedness (Sedikides & Wildschut, 2019) not only in the general population but also, as this study demonstrated, in people with dementia. Indeed, the benefits are key constituents of well-being for people with dementia (Lamont et al., 2020). However, although nostalgia may have the potential to enhance the lives of people living with dementia (Ismail et al., 2018, 2020), this will be fully realised if viable methods of integrating the emotion into a daily routine can be identified. Our study took the first step in this journey of implementation.

## Notes

1. The study was granted NHS Research Ethics Committee approval on 17^th^ July 2018 (18/WA/0198) and was subsequently ratified by the University of the West of England HAS Faculty Ethics committee on 10^th^ August 2018 (HAS.18.08.001).

2. ACORN_RCI_and_clinical_cutoff_calculator.xls, provided by the ACORN Organization Inc.

3. We used pseudonyms.
